# Breaking Through Resistance: A Comparative Review of New Beta-Lactamase Inhibitors (Avibactam, Vaborbactam, Relebactam) Against Multidrug-Resistant Superbugs

**DOI:** 10.3390/antibiotics14050528

**Published:** 2025-05-21

**Authors:** Ilias Karaiskos, Irene Galani, George L. Daikos, Helen Giamarellou

**Affiliations:** 1 First Department of Internal Medicine-Infectious Diseases, Hygeia General Hospital, 15123 Athens, Greece; e.giamarellou@hygeia.gr; 2 Infectious Diseases Laboratory, Fourth Department of Internal Medicine, National and Kapodistrian University of Athens, 12462 Athens, Greece; egalani@med.uoa.gr; 3 Second Department of Internal Medicine, Mitera General Hospital, 15123 Athens, Greece; gldaikos@gmail.com

**Keywords:** β-lactam–β-lactamase inhibitors, ceftazidime/avibactam, meropenem/vaborbactam, imipenem/cilastatin/relebactam, aztreonam/avibactam, ceftazidime/avibactam plus aztreonam, resistance mechanism, comparative efficacy, treatment failure, rapid detection

## Abstract

The introduction of new β-lactam–β-lactamase inhibitors (BLBLIs), such as ceftazidime/avibactam, meropenem/vaborbactam, and imipenem/cilastatin/relebactam, expands our therapeutic options against carbapenem-resistant Gram-negative bacteria, including those pathogens for which therapeutic options are limited. These new combinations are active against ESBL-, AmpC-, and KPC-producing Enterobacterales, with the exception of ceftazidime/avibactam, which is active in vitro against OXA-48. However, one drawback that must be taken seriously by the clinician is that they are ineffective against metallo-β-lactamases as well as *Acinetobacter baumannii*. The recent introduction of aztreonam/avibactam marks a significant advancement in our therapeutic armamentarium against metallo-β-lactamase-producing pathogens. The question to be answered is whether there is a preferred, newer BLBLI combination for the treatment of KPC-producing Enterobacterales infections. This review provides a thorough analysis of the similarities and differences between these new combinations to identify the most effective treatment options. The present review aims to provide clinicians with a detailed understanding of each BLBLI treatment option to guide the optimal use of these new agents for the effective treatment of difficult infections caused by carbapenemase-producing Enterobacterales infections. This review is based on literature retrieved from PubMed, Scopus, Web of Science, and the Cochrane Library.

## 1. Introduction

Escalating antimicrobial resistance (AMR) among Gram-negative bacteria, particularly carbapenemase-producing Enterobacterales (CPE), poses a significant global health threat due to limited therapeutic options and alarmingly high mortality rates. Among these pathogens, *Klebsiella pneumoniae*—the most clinically relevant member of Enterobacterales—has demonstrated especially concerning resistance patterns, often mediated by carbapenemase enzymes such as *K. pneumoniae* carbapenemase (KPC), oxacillinase (OXA-48-like), and various metallo-β-lactamases (MBLs) such as Verona integron-encoded metallo-β-lactamase (VIM) and New Delhi metallo-β-lactamase (NDM) [1]. Recent surveillance data indicate that resistance to carbapenems among invasive *K. pneumoniae* isolates in Europe has increased substantially, reaching alarming levels exceeding 60% in some southern and eastern European countries [2].

Historically, the management of multidrug-resistant (MDR) Gram-negative infections has relied on “revived” antimicrobials, including colistin, aminoglycosides, fosfomycin, and tigecycline. Nevertheless, the clinical effectiveness of these agents is frequently limited by toxicity profiles, suboptimal pharmacokinetics, and rising resistance rates, which complicate therapeutic decision-making and negatively impact patient outcomes [3].

The advent of novel β-lactam–β-lactamase inhibitor (BLBLI) combinations, such as ceftazidime/avibactam (CAZ-AVI), meropenem/vaborbactam (MER-VAB), imipenem/cilastatin/relebactam (IMI-REL), and aztreonam/avibactam (ATM-AVI), marks a significant advancement in the fight against these formidable pathogens [4]. Real-world studies underscore their efficacy, with CAZ-AVI demonstrating a clinical cure rate exceeding 70% against carbapenem-resistant Enterobacterales infections, significantly outperforming older antibiotic regimens [5,6]. Similarly, MER-VAB and IMI-REL have shown potent in vitro activity against KPC-producing strains, translating into reduced mortality rates and improved patient outcomes in clinical practice [7,8].

However, as with all antimicrobial agents, the clinical success of novel BLBLs is challenged by the adaptive capabilities of bacterial pathogens. Increasing reports of resistance emergence during CAZ-AVI therapy have been documented, particularly among *K. pneumoniae* isolates. Resistance mechanisms include mutations in bla_KPC_ alleles, as well as outer membrane porin alterations [9]. Furthermore, the acquisition of MBLs, such as NDM and VIM, confers intrinsic resistance to currently available BLBLI combinations, including CAZ-AVI, MER-VAB, and IMI-REL [10]. These findings point out the dynamic nature of bacterial resistance evolution and emphasize the necessity of robust antimicrobial stewardship and continuous resistance surveillance [11].

This narrative review was conducted by systematically searching PubMed, Scopus, Web of Science, and Cochrane Library databases for relevant English-language articles published up to January 2025. Keywords included: ‘β-lactam-β-lactamase inhibitors’, ‘novel β-lactamase inhibitors’, ‘ceftazidime-avibactam’, ‘meropenem-vaborbactam’, ‘imipenem-relebactam’, ‘imipenem-cilastatin-relebactam’, ‘aztreonam-avibactam’, ‘resistance mechanisms’, and ‘clinical outcomes’. We included original research, observational studies, systematic reviews, meta-analyses, and guideline statements. The reference lists of included articles were manually screened to identify additional relevant studies.

This comparative review aims to critically analyze and compare the clinical and microbiological efficacy, safety profiles, and emerging resistance mechanisms associated with the newest β-lactamase inhibitors, avibactam, vaborbactam, and relebactam in the management of MDR Gram-negative infections. By consolidating current scientific data and highlighting future challenges, we seek to provide clinicians and microbiologists with comprehensive insights necessary to optimize the use of these critical agents and combat the escalating threat of AMR effectively.

## 2. Epidemiological Data

The most recent antimicrobial resistance data available in Europe were published by the European Antimicrobial Resistance Surveillance Network (EARS-Net) in 2024 and reflect surveillance data from all European Union/European Economic Area (EU/EEA) countries collected during 2023. The epidemiology of AMR, particularly involving carbapenem-resistant *K. pneumoniae*, exhibited significant worsening across the EU/EAA from 2019 to 2023. Specifically, the estimated overall EU incidence of bloodstream infections (BSIs) caused by carbapenem-resistant *K. pneumoniae* increased by 57.5% compared to the 2019 baseline, surpassing the 2030 AMR reduction target. This rising incidence indicates a statistically significant upward trend and highlights a growing public health concern. Between 2019 and 2023, there was also a notable rise in the overall number of invasive *K. pneumoniae* isolates reported across the EU, with an 11.9% increase observed between 2022 and 2023 alone. This upward trajectory was observed across all resistance phenotypes for *K. pneumoniae* bloodstream infections [2].

Regarding *Pseudomonas aeruginosa*, a 41.1% increase in reported invasive isolates was recorded from 2019 to 2023, with a 6.4% increase between 2022 and 2023. In 2023, the highest EU incidence rates for resistant *P. aeruginosa* bloodstream infections were observed for carbapenems. Importantly, between 2019 and 2023, significant upward trends were observed in EU incidence rates for BSI caused by *P. aeruginosa* resistant to carbapenems, piperacillin/tazobactam, and ceftazidime, highlighting increased challenges in clinical management and infection control [2].

CPEs have become a significant public health concern across Europe, with KPC, OXA, and various MBLs such as VIM and NDM being the primary enzymes responsible for carbapenem resistance [12]. In Europe, the distribution of these carbapenemases varies by region. KPC-producing *K. pneumoniae* has notably become endemic in Italy and Greece, with substantial increases in carbapenem resistance observed over recent years [12,13]. OXA-48-like enzymes have also been frequently detected in several western European countries, being the most prevalent carbapenemase group in Spain and France [14]. OXA-48-like enzymes have also been present in Germany, Switzerland, The Netherlands, and the United Kingdom [14]. NDM-producing strains, while less prevalent, have been identified across Europe, and have shown an increasing trend in the last years and are often linked to patients with prior healthcare exposure in endemic regions [10,15].

The rising resistance of Gram-negative bacteria to carbapenems in Greece, as reported by the Greek System for the Surveillance of Antimicrobial Resistance, called WHONET, in 2024, represents a significant public health threat [16]. *Klebsiella pneumoniae* strains exhibit alarmingly high resistance rates, ranging up to 78%, with KPC- and MBL-type carbapenemases predominating at 66.5% and 28.6%, respectively [2,17]. However, of concern is the gradual increase in MBLs with the proportion mounting over 30% in 2022 [17,18,19]. Likewise, *Pseudomonas aeruginosa* demonstrates resistance rates up to 59%, placing Greece among the European countries with the highest burden of carbapenem-resistant infections. Recent Greek epidemiological studies have demonstrated high in vitro susceptibility rates to CAZ-AVI (100%), IMI-REL (98%), and MER-VAB (100%) [17,18,20].

## 3. Differences Between Newer Inhibitors (Avibactam, Vaborbactam, Relebactam)

The targeted and judicious use of novel BLBLIs is critical for the effective management of infections caused by MDR Gram-negative bacteria, particularly those involving carbapenemase-producing strains. Recent advances have introduced several novel BLBLIs, notably avibactam, vaborbactam, and relebactam, each characterized by distinct inhibitory spectra, mechanisms of action, and clinical profiles. Avibactam, a non-β-lactam diazabicyclooctane (DBO) inhibitor, demonstrates potent inhibition of Ambler class A (e.g., KPC), class C (AmpC), and certain class D (OXA-48-like) β-lactamases. Vaborbactam, a cyclic boronic acid inhibitor, is uniquely tailored for robust activity specifically against serine carbapenemases, particularly KPC enzymes, but lacks effectiveness against MBLs. Relebactam, another DBO derivative structurally related to avibactam, effectively inhibits class A (including KPC) and class C β-lactamases but has limited activity against class D carbapenemases and no efficacy against MBLs [4,21].

In an effort to delineate the key differences among the three novel β-lactamase inhibitors, avibactam, vaborbactam, and relebactam, and to facilitate optimal therapeutic decision-making, the following essential considerations should be highlighted.

### 3.1. General Management Recommendations

When selecting an appropriate BLBLI combination for the empirical treatment of severe nosocomial infections, clinicians should consider the following factors [22,23].

1. Suspected or confirmed pathogen—The selection should be guided by local epidemiology and known resistance mechanisms, as well as the presence of carbapenemases.
2. Severity and site of infection—The pharmacokinetic/pharmacodynamic (PK/PD) properties of the chosen agent should align with the infection’s anatomical site and severity.
3. History of prior MDR infections—A previous infection with an MDR pathogen within the past six months may necessitate a more targeted approach.
4. Previous exposure to carbapenems and other antibiotics—Recent treatment with carbapenems, quinolones, or other broad-spectrum antibiotics within the past three months may influence resistance selection and treatment efficacy.
5. History of ICU admission—Prior hospitalization in an ICU is a known risk factor for infections caused by carbapenem-resistant organisms.
6. Hospital and regional epidemiology—The prevalence of carbapenemase-producing Enterobacterales in the hospital should be factored into empirical antibiotic selection.
7. Known colonization with carbapenemase-producing bacteria—Patients with documented colonization may require preemptive adjustments to empiric therapy.
8. Recent hospitalization in a ward with carbapenemase-producing outbreaks—Close contact with patients infected or colonized with carbapenemase-producing bacteria increases the likelihood of acquiring a resistant strain.
9. Transfer from long-term care facilities or rehabilitation centers—Patients transferred from these settings often have prolonged antibiotic exposure and a higher risk of colonization with MDR pathogens.
10. Hospital-acquired septic shock—In cases of septic shock, early and aggressive antimicrobial intervention with broad-spectrum agents, including BLBLIs, is critical for improving patient outcomes.

These considerations indicate the necessity of an evidence-based, individualized approach when selecting BLBLIs for empirical treatment, ensuring both optimal patient outcomes and the preservation of antimicrobial efficacy in the long term.

All three BLBLIs, avibactam, vaborbactam, and relebactam, demonstrate no activity against *Acinetobacter baumannii*. In vitro studies have confirmed that these novel BLBLI combinations exhibit high efficacy against extended-spectrum β-lactamase (ESBL)-, AmpC-, and KPC-producing strains. However, CAZ-AVI is uniquely active against *OXA-48*-producing *K. pneumoniae*, whereas all three agents lack efficacy against MBL-producing strains, including those harboring NDM, VIM, or IMP enzymes [4,21]. Due to their ineffectiveness against MBL-expressing pathogens, alternative treatment strategies, such as combination regimens incorporating aztreonam with CAZ-AVI or cefiderocol, are being explored to counteract these highly resistant infections [24].

Notably, relebactam lacks in vitro activity against *Proteus* spp., *Providencia* spp., and *Morganella* spp. strains. Therefore, in healthcare settings where these pathogens are prevalent, empirical therapy with IMI-REL should be avoided, and alternative combinations such as CAZ-AVI or MER-VAB should be considered when MBL production is not a factor [4,21].

Regarding *Pseudomonas aeruginosa*, strains resistant to carbapenems or all β-lactams but lacking MBL production are typically susceptible to CAZ-AVI and IMI-REL, whereas MER-VAB is generally ineffective [4,21,24]. However, in cases where *P. aeruginosa* produces β-lactamases from the Guiana extended-spectrum β-lactamase (GES) family, resistance extends not only to ceftolozane/tazobactam but also to IMI-REL. At least in vitro, such strains remain susceptible to CAZ-AVI [25,26].

The comparative in vitro activity of these newer BLBLIs against carbapenem-resistant Gram-negative bacteria is summarized in Table 1 [4,21,24,25]. These findings support the importance of pathogen-specific treatment strategies and the need for ongoing surveillance to optimize therapeutic decision-making.

For adult patients with normal renal function, the standard dosing regimens are as follows: CAZ-AVI at 2.5 g intravenously (IV) every 8 h [27], MER-VAB at 4 g IV every 8 h [28], and IMI-REL at 1.25 g IV every 6 h [29]. Both MER-VAB and CAZ-AVI are suggested to be administered via prolonged infusion (>3 h) to optimize pharmacokinetics and pharmacodynamics, improving drug exposure and time above the minimum inhibitory concentration (T > MIC) [5,28]. In contrast, the infusion time for IMI-REL should not exceed 30 min due to pharmacokinetic properties specific to imipenem [29]. ATM-AVI, which is indicated for infections caused by MBL-producing Enterobacterales, has a recommended dosing regimen of 2 g/0.67 g as a loading dose, followed by 1.5 g/0.5 g IV every 6 h in a prolonged 3-h infusion to maximize efficacy [30].

### 3.2. Real-Life Clinical Studies of Novel BLBLI Agents

In terms of therapeutic efficacy, all three agents have drastically improved outcomes for patients with KPC-producing Enterobacterales infections compared to legacy regimens. They achieve high cure rates in BSI, pneumonia, complicated intra-abdominal infections (cIAI), and complicated urinary tract infections (cUTI) caused by these MDR pathogens. Mortality in severe carbapenemase-producing Enterobacterales (CRE) infections has dropped substantially with the availability of these drugs, often turning a previously untreatable infection into a treatable one. Where polymyxin-based regimens might have yielded barely 30–40% survival in some studies, the new BLBLIs therapies have pushed survival well above 70–80% in similar cohorts. This efficacy, coupled with far better safety profiles, makes them the preferred choices [5,6,31,32,33,34,35].

#### 3.2.1. Ceftazidime/Avibactam

CAZ-AVI has shown robust clinical efficacy and reduced mortality in the treatment of infections caused by CRE, particularly those due to KPC-producing strains. Across several real-world studies, CAZ-AVI consistently demonstrated superior clinical outcomes compared to older, nephrotoxic agents like colistin [5,6,31,32,33,34,35]. In a large multicenter cohort of 147 patients with CRE infections (140 KPC and 7 OXA-48 producers), CAZ-AVI treatment resulted in 14-day and 28-day mortality rates of 9% and 20%, respectively. Importantly, among patients with BSIs due to KPC-producing *K. pneumoniae*, those treated with CAZ-AVI experienced significantly lower 28-day mortality compared to matched patients receiving alternative therapies (18.3% vs. 40.8%, *p* = 0.005) [5]. Similarly, in a comparative cohort study, CAZ-AVI therapy led to significantly higher microbiological eradication (94.3% vs. 67.7%), clinical cure rates (80.5% vs. 52.8%), and 28-day survival (85.4% vs. 61.1%) relative to non-CAZ-AVI regimens, with fewer relapses (2 vs. 12, *p* = 0.042). CAZ-AVI use was an independent predictor of survival and clinical cure in multivariate analysis [31]. In a broader cohort of 577 patients treated with CAZ-AVI—either as monotherapy or in combination—30-day all-cause mortality was 25%, with no significant difference between monotherapy and combination treatment. Factors associated with increased mortality included septic shock, neutropenia, lower respiratory tract infection, and CAZ-AVI dose adjustment for renal impairment. Conversely, administration of CAZ-AVI by prolonged infusion was associated with improved survival (*p* = 0.006) [6]. In a multicenter retrospective study from Spain, CZA-AVI treatment was associated with significantly lower 30-day mortality compared to best available therapy (BAT) in patients with CPE infections. The benefit was particularly pronounced in patients with INCREMENT-CPE scores >7 points, where mortality was 21.9% with CZA-AVI versus 46.9% with BAT (*p* = 0.004). Multivariate analysis confirmed that CZA-AVI was an independent predictor of survival (OR 0.41, 95% CI 0.20–0.80; *p* = 0.01), while high INCREMENT-CPE scores remained a significant risk factor for mortality [32].

Direct comparisons with polymyxin-based regimens further highlight CAZ-AVI’s favorable profile. In a study comparing 38 patients treated with CAZ-AVI versus 99 with colistin-based therapies, 30-day mortality was significantly lower in the CAZ-AVI group (9% vs. 32%, *p* = 0.001). CAZ-AVI was associated with a higher probability of clinical success and fewer adverse outcomes, including a markedly lower rate of nephrotoxicity, which was more common in colistin-containing regimens [33]. Similarly, in a multicenter observational study, initial treatment with CZA-AVI was associated with significantly improved 30-day outcomes compared to colistin, with a 64% probability of a better clinical outcome and substantially lower all-cause hospital mortality in patients with *K. pneumoniae* carbapenemase–producing CRE infections [34]. The introduction of CAZ-AVI appears to have significantly improved outcomes in patients with KPC-producing CRE. In this cohort of 426 patients, 30-day mortality did not significantly differ between patients appropriately treated with CAZ-AVI for carbapenem-resistant *K. pneumoniae* BSI and those treated for carbapenem-susceptible *K. pneumoniae* BSI, suggesting that carbapenem resistance per se is no longer a major determinant of mortality in settings where KPC-producing CRE is predominant [36]. Furthermore, the attributable mortality for KPC-CRE BSI was as low as 5% in the Advancing knowLedge on Antimicrobial Resistant Infections COllaboration (ALARICO) Network, further supporting the notion that timely and appropriate use of CAZ-AVI can effectively mitigate the adverse outcomes historically associated with CRE infections [37].

#### 3.2.2. Meropenem/Vaborbactam

Real-world clinical experiences support the efficacy of MER-VAB for treating infections caused by CRE [7,38,39,40,41,42]. Observational studies on MER-VAB efficacy reported clinical success rates between 60 and 75%, with 30-day mortality generally ranging from 15–30%, even among critically ill populations. Given its activity against CAZ-AVI-resistant KPC variants and its favorable PK/PD properties, MER-VAB emerges as a strong first-line option for treating severe KPC-CRE infections [43].

A prospective observational study involving 20 patients with CRE infections treated with MER-VAB reported a 30-day clinical success rate of 65%, with a corresponding survival rate of 90%. However, microbiologic failures occurred in 35% of patients within 90 days, including one patient who developed a recurrent infection caused by a meropenem/vaborbactam-non-susceptible *K. pneumoniae* isolate harboring an ompK36 porin mutation [38]. Additionally, in a larger multicenter observational study of 104 patients treated with MER-VAB, clinical success was observed in 77%, and the overall 30-day mortality rate was 15.4%. Notably, among patients specifically infected with KPC-producing *K. pneumoniae*, clinical success and 30-day mortality rates were 82% and 11.5%, respectively [7]. In line with these results, in another multicenter real-world study with 126 patients, MER-VAB demonstrated favorable clinical outcomes and low 30-day mortality (18.3%) in patients with Gram-negative infections, predominantly caused by carbapenem-resistant Enterobacterales, including *K. pneumoniae* (42.1%). Among patients infected with CR-*K. pneumoniae*, early initiation of MER-VAB (within 48 h) significantly improved outcomes, highlighting its critical role in treating these high-risk, multidrug-resistant infections [39]. Moreover, a multicenter cohort study involving 342 adult patients evaluated MER-VAB predominantly for BSI (n = 172) and nonbacteremic infections (n = 170), including lower respiratory tract infections, cUTI, and other infection sites. In this study, 62.3% of patients received MER-VAB monotherapy, and 37.7% were treated with MER-VAB in combination with another active antibiotic (commonly fosfomycin, tigecycline, or gentamicin). The overall 30-day mortality rate was 31.6%. Independent predictors of increased mortality included septic shock at infection onset, Charlson comorbidity index ≥3, dialysis dependence, concomitant COVID-19 infection, and an INCREMENT score ≥8. Early initiation of MER-VAB therapy within 48 h from infection onset was initially associated with reduced mortality, although this association was not sustained after adjusting for propensity scores related to combination therapy [42]. Collectively, these findings highlight the clinical value of MER-VAB as an effective therapeutic option for CRE infections, emphasizing the importance of early initiation and appropriate source control to improve patient outcomes [7,39,42].

#### 3.2.3. Imipenem/Cilastatin/Relebactam

IMI-REL has been evaluated in limited real-world studies for its efficacy against CRE and mainly for difficult-to-treat resistant (DTR) *P. aeruginosa* infections in critically ill patients [8,44,45,46]. A single-center case series reported successful treatment of 10 patients with complicated infections caused by CRE and DTR *P. aeruginosa*, achieving clinical cure in all cases without adverse effects [44]. Additionally, a multicenter observational study assessed 14 patients receiving IMI-REL for DTR *P. aeruginosa* infections. Clinical efficacy and microbiological success were observed in 64.3% of cases, with a 30-day all-cause mortality rate of 42.9% [8]. Consistent with these findings, in a multicenter, retrospective case series of 21 patients, IMI-REL was primarily used to treat DTR *P. aeruginosa* and carbapenem-resistant *K. pneumoniae* infections. Clinical cure was achieved in 62% of patients, and the 30-day all-cause mortality rate was 33%. Most infections were respiratory tract infections (52%), and IMI-REL therapy was often selected when no other active agent was available. Importantly, resistance to IMI-REL developed in only one patient during treatment [45]. Furthermore, a real-world evaluation across multiple U.S. medical centers analyzed 160 patients treated with IMI-REL for multidrug-resistant Gram-negative infections. The study found that 85.4% of the pathogens were carbapenem-non-susceptible, with *P. aeruginosa* being the most frequently targeted organism (72.2%). Clinical success was achieved in a significant proportion of cases (70%, 106/151), indicating the potential effectiveness of IMI-REL in managing severe infections caused by resistant pathogens [46]. These findings suggest that IMI-REL may be a viable treatment option for critically ill patients with CRE and DTR *P. aeruginosa* infections. However, the observed mortality rates underscore the severity of these infections and the need for further large-scale studies to better define the role of IMI-REL in this clinical real-life context and to optimize treatment strategies for these high-risk patients.

### 3.3. Comparative Efficacy and Key Considerations

Direct comparisons between CZA-AVI, MER-VAB, and IM-REL are limited [47,48,49], but available evidence suggests all three have comparable clinical effectiveness against KPC-producing Enterobacterales [3,4,21,34,47,48,49]. A retrospective multicenter study (2015–2018) compared CAZ-AVI and MER-VAB in 131 patients with serious CRE infections. Clinical success was similar: 62% with ceftazidime/avibactam vs. 69% with MER-VAB (*p* = 0.49). There were no significant differences in 30-day or 90-day mortality or adverse events between the two groups. Interestingly, clinicians tended to combine other antibiotics with CAZ-AVI more often than with MER-VAB (61% of CAZ-AVI patients received combination therapy vs. only 15% with MER-VAB). Despite more frequent combination use, CAZ-AVI outcomes were not superior, reinforcing that MER-VAB monotherapy was equally effective in this cohort. One notable finding was that the emergence of resistance occurred in 3 of 15 patients who had recurrent infections after CAZ-AVI monotherapy, whereas no resistance developed among those treated with MER-VAB [48]. A recent multicenter retrospective cohort study compared the outcomes of MEV-VAB and CZA-AVI among adults hospitalized with serious infections in the United States from 2019 to 2021. The study included 2775 patients, with 455 receiving MER-VAB and 2320 receiving CZA-AVI. After adjusting for confounders, treatment with MER-VAB was linked to reduced hospital mortality (16.5% vs. 20.6%, *p* = 0.021) and a shorter ICU length of stay following infection onset (19.6 days vs. 22.4 days, *p* = 0.042) compared to CAZ-AVI in adults hospitalized with serious infections, primarily pneumonia and cUTI. Although both agents were used relatively late (median 5–6 days after infection onset) and predominantly in critically ill patients, mechanical ventilation was required less frequently with MER-VAB (35% vs. 41%, *p* = 0.010). MER-VAB also showed a trend toward lower *Clostridioides difficile* infection incidence, although this was not statistically significant. While clinical cure and mortality were broadly similar, these findings suggest that MER-VAB may offer a slight survival advantage and shorter critical care needs compared to CAZ-AVI, potentially due to stronger activity against KPC-producing strains and lower rates of emergence of resistance. However, the study’s observational design necessitates cautious interpretation, and further research is warranted to confirm these results. However, overall, both agents achieved high cure rates in the study [49]. No head-to-head studies comparing IMI-REL to either CAZ-AVI or MER-VAB have been completed to date. Given the lack of direct comparative trials, current IDSA guidelines list all three as preferred options for KPC-producing CRE infections [24].

The CACTUS study was a large, multicenter, matched, retrospective cohort study evaluating the comparative effectiveness of CZA-AVI versus ceftolozane/tazobactam for the treatment of DTR *Pseudomonas aeruginosa* infections in the United States, including pneumonia (83%) and bacteremia (17%), across 28 hospitals. Among 420 critically ill patients, clinical success at 30 days was significantly higher with ceftolozane/tazobactam compared to ceftazidime/avibactam (61% vs. 52%; adjusted OR 2.07, 95% CI 1.16–3.70), with the benefit mainly driven by better outcomes in pneumonia cases (63% vs. 51%; adjusted OR 2.34, 95% CI 1.23–4.47). In contrast, no significant difference in clinical success was observed among patients with bacteremia (51% vs. 57%; adjusted OR 0.76, 95% CI 0.23–2.57). Despite differences in clinical success, 30-day and 90-day mortality rates were similar between groups (~23–24% and ~37–38%, respectively), and treatment-emergence of resistance developed in over 20% of patients in both arms. These findings suggest that ceftolozane/tazobactam may offer a clinical advantage over CZA-AVI in pneumonia due to multidrug-resistant *P. aeruginosa*, whereas outcomes in bacteremia appear comparable, underscoring the need for further optimization of therapy in this critically ill population [50].

When selecting among novel BLBLI combinations, CAZ-AVI, MER-VAB, and IMI-REL, clinicians must carefully consider several factors, including the specific resistance mechanisms of the infecting pathogen, infection site, pharmacokinetic properties, patient-specific comorbidities, and institutional availability or experience with these agents [3,4,21,24,47,51].

Spectrum considerations play a critical role in therapeutic decisions. If the organism produces an OXA-48-type carbapenemase, CZA-AVI is uniquely effective due to avibactam’s potent inhibitory activity against OXA-48 enzymes. Conversely, none of these agents are active against MBL-β-lactamase-producing pathogens, such as those harboring NDM enzymes, necessitating alternative treatments, typically cefiderocol or aztreonam-based regimens. For pathogens producing KPC or AmpC enzymes, all three agents retain activity [3,4,21]. Notably, IMI-REL offers an additional advantage for imipenem-resistant *P. aeruginosa* infections, particularly when resistance is driven by AmpC derepression or efflux pump mechanisms [51]. In contrast, MER-VAB primarily targets KPC-producing Enterobacterales and does not significantly enhance activity against resistant *Pseudomonas* isolates [21,43].

Pharmacokinetic and patient-specific factors further influence the choice among these agents. All three combinations require dose adjustments in patients with renal impairment, including those undergoing hemodialysis [52]. Dosage adjustment of novel BLBLIs is depicted in Table 2 [27,28,29,30,52]. Notably, IMI-REL is administered at more frequent intervals (every 6 h), which may pose logistical challenges in outpatient scenarios or settings employing numerous infusion strategies [29,51]. MER-VAB requires extended infusions, potentially impacting intravenous line availability in inpatient settings, and also delivers a comparatively higher sodium load, a consideration for patients with heart failure or fluid restrictions [28]. Allergy profiles may also influence selection, as ceftazidime is a cephalosporin and thus may have a different cross-reactivity risk compared with carbapenems such as meropenem and imipenem [28,29,53]. Therefore, in cases of penicillin allergy, CAZ-AVI is considered the BLBLI of choice with the less possibility of allergic reactions. Additionally, clinicians must weigh the neurological side-effect profiles of these β-lactams, as imipenem carries a recognized risk of seizure precipitation in susceptible patients, while meropenem is typically associated with a lower neurological risk [28,29,54].

Institutional availability and clinical experience also significantly impact therapeutic choices. Among the three agents, CAZ-AVI has been commercially available the longest, resulting in broader clinical familiarity, including in pediatric populations. MER-VAB and IMI-REL, while newer, are increasingly incorporated into hospital formularies, reflecting growing recognition of their utility against carbapenem-resistant infections, particularly those caused by KPC-producing organisms [55,56,57]. A survey conducted among U.S. hospitals in 2018 indicated widespread inclusion of CAZ-AVI and MER-VAB, pointing out their established role as frontline therapies against carbapenem-resistant Enterobacterales [55]. Between 2015 and 2021, CZA-AVI utilization in the Veterans Affairs Healthcare System rose substantially, with an estimated annual increase of 52.3% in days of therapy per 1000 bed days (95% CI, 12.4–106.4%), highlighting its expanding use against multidrug-resistant Gram-negative infections [56]. In a large retrospective cohort study encompassing 832 hospitals across the United States from June 2022 to May 2023, next-generation Gram-negative antibiotics were prescribed in 0.25% of antibiotic-treated admissions. Among these, ceftolozane/tazobactam (42.6%) and CZA-AVI (37.5%) were the most frequently utilized agents, while cefiderocol (10.9%), MER-VAB (4.7%), and IMI-REL (0.9%) demonstrated a more limited uptake. Notably, 46% of new antibiotic prescriptions were initiated within the first three days of admission, predominantly for severe infections such as sepsis (76.2%), pneumonia (45.8%), and cUTI (38.7%) [57]. While IMI-REL’s clinical adoption has been slower initially, accumulating real-world evidence is likely to facilitate broader acceptance and utilization in clinical practice. A comprehensive comparison of novel BLBLIs is illustrated in Table 3 [3,4,5,6,7,8,27,28,29,30,31,32,33,34,35,36,37,38,39,40,41,42,43,44,45,46,47,48,49,50,58,59,60].

### 3.4. Management Recommendations for MBL-Producing Strains

In cases of infection caused by MBL-producing strains, the treatment of severe nosocomial sepsis necessitates the use of aztreonam in combination with avibactam [4,10,21]. This regimen, recently approved by the European Medicines Agency (EMA), is known as aztreonam/avibactam (ATM-AVI) [30]. ATM-AVI is an antibiotic approved by the EMA for the treatment of adults with cIAI, hospital-acquired pneumonia (HAP), including ventilator-associated pneumonia (VAP), cUTI, including pyelonephritis, as well as infections caused by aerobic Gram-negative bacteria in patients with limited treatment options [30]. Similarly, the U.S. Food and Drug Administration (FDA) has approved ATM-AVI for the treatment of adults with cIAI when limited or no treatment options are available [58].

Avibactam effectively inhibits Ambler class A and C β-lactamases, as well as certain class D enzymes. However, it does not inhibit MBLs. The combination of avibactam with the monobactam aztreonam provides potent activity against MBL-producing carbapenem-resistant Gram-negative bacteria (CR-GNB). This efficacy is due to the fact that MBLs are incapable of hydrolyzing aztreonam, which remains structurally intact. Additionally, avibactam enhances the activity of aztreonam by protecting it from degradation by coexisting serine β-lactamases, which are frequently present in CR-GNB. Thus, for Enterobacterales strains exhibiting multiple resistance mechanisms involving VIM or NDM-type metallo-β-lactamases in combination with co-expression of ESBLs, KPC, OXA-48, or AmpC β-lactamases, the combination therapy of ATM-AVI represents an effective therapeutic approach [4,10,21].

The approval of ATM-AVI was primarily based on data derived from two phase 3 clinical studies, REVISIT and ASSEMBLE [59,60]. In the REVISIT trial, ATM-AVI (in combination with metronidazole for cIAI) was compared with meropenem (±colistin) for the treatment of infections caused by Gram-negative pathogens, including cIAI, HAP, and VAP. The study outcomes demonstrated comparable rates of clinical cure between both treatment arms, with no statistically significant difference in 28-day mortality [59].

In the ASSEMBLE trial, ATM-AVI was compared with the best available therapy for the treatment of infections caused by MBL-producing Gram-negative organisms. Although this study was terminated prematurely due to enrollment challenges, the data indicated that clinical cure was achieved in 42% (5/12) of patients treated with ATM-AVI, while none (0/3) of the patients receiving best available therapy achieved clinical cure [60].

Overall, the clinical use of ATM-AVI for infections caused by extensively drug-resistant pathogens, particularly those producing MBLs, is primarily supported by limited data from the REVISIT study (422 patients with cIAI or HAP/VAP, including 17 patients infected with carbapenem-resistant organisms) [59], and the ASSEMBLE trial (12 out of 15 patients treated with ATM-AVI) [60]. Despite the limited data available, ATM-AVI represents a promising therapeutic option for managing infections due to MBL-producing Gram-negative bacteria.

Until this combination becomes widely available, an alternative regimen involving CAZ-AVI combined with aztreonam (ATM) can be utilized. For optimal efficacy, both agents should be administered simultaneously via the same intravenous line [24]. This recommendation is supported by robust clinical studies demonstrating high rates of clinical and microbiological success [61,62].

The combination of CAZ-AVI plus aztreonam (CZA-AVI+ATM) has emerged as a promising therapeutic option for BSI caused by MBL-producing Enterobacterales, parti- cularly NDM and VIM producers. In a large prospective study of 343 patients from 2019 to 2022, CZA-AVI+ATM was the most commonly used regimen for treating infections caused by MBL-producing Enterobacterales, administered in 62.7% of patients. Compared to colistin-containing regimens, CZA-AVI+ATM was independently associated with a significant reduction in 30-day mortality (adjusted hazard ratio [aHR], 0.39; 95% CI, 0.18–0.86; *p* = 0.02), with synergy between CAZ-AVI and ATM demonstrated in 99.7% of tested isolates. Other treatment regimens included cefiderocol-containing therapies (9.6%), colistin-containing regimens (7.6%), and other active antibiotics (10.8%). Although cefiderocol showed some promise, its association with mortality reduction was not statistically significant, likely due to the limited number of cases. Importantly, patients receiving colistin had substantially higher rates of adverse events, particularly acute kidney injury, compared to those treated with CZA-AVI+ATM [61]. Another study of 102 patients with BSI due to NDM- or VIM-producing Enterobacterales found that CAZ-AVI+ATM was associated with lower 30-day mortality (*p* = 0.01), reduced clinical failure on day 14 (*p* = 0.002), and shorter hospital stays (*p* = 0.007) compared to other active antibiotics (OAAs). Compared with OAAs, treatment with the CZA-AVI+ATM combination conferred a significant therapeutic advantage, demonstrating an approximately 60% reduction in mortality risk even after adjustment for baseline characteristics and propensity score matching. Furthermore, patients receiving CZA-AVI+ATM exhibited the lowest rates of clinical failure by day 14 following BSI onset and experienced a significantly shorter length of hospital stay (LOS) [62]. These findings support the use of CZA-AVI+ATM as the preferred therapeutic option for managing infections caused by MBL-producing Enterobacterales, given its demonstrated survival benefit and reduced clinical failure rates [24,61,62]. In the absence of widespread clinical availability and real-world assessment of the newly approved ATM-AVI combination, CZA-AVI+ATM serves as a critical bridging therapy, providing an essential and effective treatment strategy to address the urgent clinical need for managing MBL-producing Gram-negative infections. Until broader access and robust clinical validation of ATM-AVI are achieved globally, the CZA-AVI+ATM combination remains a reliable and evidence-based approach for these challenging infections.

## 4. Development of Resistance

Resistance to novel β-lactamase inhibitors primarily arises through the following mechanisms [63].

- Reduced porin expression—Decreased outer membrane permeability limits intracellular drug penetration, reducing the efficacy of β-lactams.
- Increased expression of carbapenemases and/or mutations in β-lactamase enzymes—Enhanced enzymatic activity contributes to β-lactam degradation and resistance development.
- Overexpression of efflux pumps—Increased antibiotic efflux mechanisms lower intracellular drug concentrations, diminishing therapeutic effectiveness.
- A combination of the above mechanisms—The interplay of multiple resistance pathways further compromises the effectiveness of BLBLI therapy, leading to high-level resistance.

### 4.1. Development of Resistance to Ceftazidime/Avibactam

Resistance to CAZ-AVI primarily arises through mutations in KPC-2 and KPC-3 carbapenemases. By 2021, 73 KPC variants had been identified, including 44 KPC-2 and 29 KPC-3 variants [63,64]. In contrast, resistance to the other two β-lactamase inhibitors, MER-VAB and IMI-REL, is more commonly associated with mutations in porins and efflux pumps [63], the National Center for Biotechnology Information (NCBI) gene reference list reports the existence of 131 KPC variants resistant to inhibitors through March 2025 [65].

The development of CAZ-AVI resistance due to KPC-2 or KPC-3 mutations typically occurs during treatment and is primarily linked to mutations in the Ω-loop of the KPC enzyme. Notably, the D179Y (Asp179Tyr) mutation—where aspartic acid at position 179 is replaced by tyrosine—enhances the hydrolytic activity of KPC against ceftazidime while simultaneously reducing the inhibitory effect of avibactam [63]. Interestingly, this mutation restores meropenem susceptibility by decreasing the minimum inhibitory concentration (MIC) by 2- to 9-fold. However, further clinical validation of this phenomenon is required [63,64,66].

In Greece, an alternative mechanism of resistance to ceftazidime/avibactam (CAZ-AVI) has been sporadically reported. This resistance mechanism involves the production of VEB-25, a plasmid-encoded extended-spectrum β-lactamase (ESBL) harboring the Lys234Arg mutation, which confers resistance through decreased susceptibility to inhibition by avibactam [67,68,69,70]. The detection of VEB-25 in a clinical isolate from Switzerland, originating from a patient previously hospitalized in a Greek healthcare facility, suggests potential undetected dissemination of this resistance mechanism within Greece [71]. According to recently published data from Greek hospitals, a total of 15 patients colonized with VEB-25-producing isolates resistant to CAZ-AVI have been identified. Among these patients, eight subsequently developed infections attributed to these resistant organisms; however, systematic epidemiological surveillance remains insufficient [67,68,69,70]. Notably, resistance mediated by VEB-25 to CAZ-AVI has not demonstrated a strong correlation with prior exposure to this antibiotic combination in most reported cases [67,68,69,70]. Importantly, while VEB-25 confers resistance to CAZ-AVI, it does not affect susceptibility to MER-VAB or IMI-REL [68,70].

A systematic literature review by Di Bella et al. summarized clinical cases of CAZ-AVI resistance and found that strains resistant to CAZ-AVI despite no prior exposure were primarily linked to VEB-25 production, KPC variants (e.g., KPC-8 and KPC-23), or an increased copy number of the *bla*KPC-2 or *bla*KPC-3 genes in combination with non-functional porins OmpK35 and OmpK36. These findings emphasize the diverse mechanisms underlying CAZ-AVI resistance and highlight the need for continued surveillance and antimicrobial stewardship to mitigate the spread of resistant Enterobacterales [72].

### 4.2. Development of Resistance to Meropenem/Vaborbactam and Imipenem/Cilastatin/Relebactam

MER-VAB resistance appears to involve a combination of KPC overproduction and mutations in the OmpK35 and OmpK36 porin genes [73]. The OmpK35 and OmpK36 porins are crucial for vaborbactam function, and their reduction or mutation is often associated with resistance development. Most notably, deficiencies or amino acid duplications in OmpK36 significantly reduce vaborbactam efficacy [73]. Additionally, reduced expression of porin genes due to the loss of transcription factors has been shown to further diminish vaborbactam’s effectiveness [74].

Mutations in OmpK35 and OmpK36 frequently coexist with other resistance mechanisms, including increased KPC expression [75]. A clinical case of resistance development following MER-VAB treatment in a *K. pneumoniae* strain producing KPC-3 was linked to increased copy numbers of the *bla*KPC gene alongside OmpK35 and OmpK36 disruption. This strain also exhibited resistance to the IMI-REL combination, highlighting a potential cross-resistance mechanism [76]. In vivo, the development of MER-VAB resistance appears to be lower compared to CAZ-AVI, particularly in strains with MICs ≤ 4/8 mg/L [63,76]. However, this may be partly attributed to the longer clinical use of CAZ-AVI, which has provided more opportunities for resistance selection.

The mechanism of IMI-REL resistance remains the least studied. Interestingly, resistance to IMI-REL during treatment in KPC-producing strains has not been reported [77]. However, current data suggest that resistance is primarily associated with carbapenemase production that is not inhibited by relebactam [78], mutations in OmpK35 and OmpK36 [20], and overexpression of *bla*KPC [76]. IMI-REL resistance emerged in 5 patients with MDR *Pseudomonas aeruginosa* pneumonia after 10–28 days of treatment, driven by mutations in MexAB-OprM and MexEF-OprN efflux pumps. Testing with an efflux inhibitor restored drug susceptibility in most cases, highlighting efflux-mediated resistance as a key mechanism. While resistance to IMI-REL among KPC-producing organisms has not been commonly reported, these findings show that *P. aeruginosa* can rapidly develop resistance through alternative mechanisms, emphasizing the need for careful stewardship of novel BLBLI therapies [79].

Recent surveillance data evaluating the efficacy of novel BLBLI combinations against CRE have highlighted the emergence of resistance across agents. According to a nationwide multicenter observational study conducted in Italy during 2022–2023, resistance rates were reported as 6.1% for CAZ-AVI, 4.9% for MER-VAB, and 3.0% for IMI-REL [80]. Complementary data from a multicenter cohort involving 17 ICUs in Greece between 2021 and 2024 demonstrated similar findings, with resistance rates of 7.0% for CZA-AVI, 3.0% for MER-VAB, and 3.5% for IMI-REL [81]. These results emphasize the ongoing need for antimicrobial stewardship and resistance surveillance, even for recently introduced agents.

### 4.3. Development of Resistance to Aztreonam/Avibactam

Strains resistant to ATM-AVI have been documented, with one study reporting that 15% of *Escherichia coli* strains producing NDM exhibit resistance to this combination [82]. A well-recognized mechanism contributing to reduced susceptibility in *E. coli* involves amino acid insertions at position 333 of penicillin-binding protein 3 (PBP3), leading to decreased affinity for aztreonam. Additionally, strains exhibiting high-level AmpC β-lactamase expression, particularly those producing CMY-42, have demonstrated clinically significant resistance to ATM-AVI [82].

**Figure 1:**
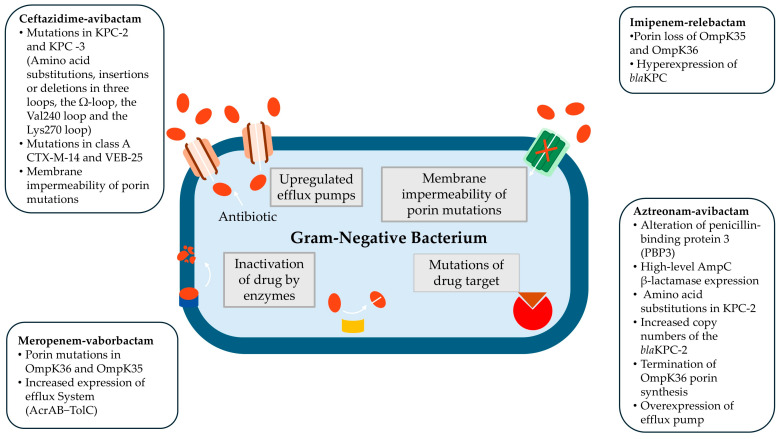
The most common mechanisms of resistance to novel BLBLIs from Gram-negative bacteria [63,64,70,72,73,74,75,76,82,83].

However, in *K. pneumoniae*, PBP3 modifications are not commonly observed, and the precise mechanisms underlying resistance to ATM-AVI remain unclear. A recent study reported high-level resistance to ATM-AVI in *K. pneumoniae* associated with a combination of factors, including amino acid substitutions in KPC-2, increased copy numbers of the *bla*_KPC-2_ gene, premature termination of OmpK36 porin synthesis, and overexpression of efflux pumps [83]. These findings highlight the complexity of resistance evolution and the necessity for ongoing surveillance and molecular characterization to optimize treatment strategies. The most common mechanisms of resistance to novel BLBLIs from Gram-negative bacteria are depicted in Figure 1 [63,64,70,72,73,74,75,76,82,83].

### 4.4. Risk Factors for Treatment Failures and Resistance

Certain clinical conditions, notably severe nosocomial pneumonia and renal replacement therapy (RRT) have been identified as significant risk factors for treatment failure and the development of resistance, particularly in patients receiving CAZ-AVI [84]. Accumulating clinical evidence indicates that CAZ-AVI may be associated with suboptimal outcomes in lower respiratory tract infections (LRTIs) caused by CRE [5,6,49,50,84]. In one clinical cohort, pneumonia was the most frequent infection type; however, the clinical success rate in this subgroup was only 36%, substantially lower than the success rates observed for urinary tract infections (88%) and primary BSI (75%) [84]. Similarly, data from a large Italian cohort identified lower respiratory tract infections as an independent predictor of mortality in patients treated with CAZ-AVI [5], while findings from a multicenter Greek study revealed a 38% mortality rate among patients with CRE pneumonia treated with CAZ-AVI, compared to 18% in those with bloodstream infections [6].

A plausible explanation for this disparity may lie in the PK/PD properties of CAZ-AVI in pulmonary tissue. Although ceftazidime and avibactam achieve measurable concentrations in the epithelial lining fluid (ELF) in the animal model [85] and of healthy volunteers [86], the average ELF penetration is approximately 30% of plasma concentrations. In contrast, meropenem and vaborbactam demonstrate markedly higher ELF penetration, reported at approximately 65% and 79%, respectively [87]. These pharmacokinetic differences may contribute to the diminished clinical efficacy of CAZ-AVI in pulmonary infections. Given the relatively lower pulmonary exposure of CAZ-AVI and the associated poor clinical outcomes in pneumonia, its use in treating CRE-associated LRTIs should be approached with caution. In such cases, alternative BLBLI combinations with superior pulmonary distribution, such as MER-VAB, may offer improved therapeutic efficacy and should be considered.

In addition to pneumonia, RRT has also emerged as an independent predictor of CAZ-AVI treatment failure and resistance development [84]. Subtherapeutic drug exposures during RRT may compromise antimicrobial efficacy and foster resistance selection. Therefore, in patients undergoing RRT and particularly in scenarios with elevated effluent flow rates or for patients with significant residual renal function, dose reductions of CAZ-AVI should be avoided to ensure adequate drug concentrations are achieved and maintained. Therefore, the priority should be to optimize drug exposures with robust dosages of CZA-AVI (i.e., 2.5 g administered every 8 h as extended infusions) rather than dose reduction for patients receiving CRRT, guided by therapeutic drug monitoring where available, may be warranted in high-risk populations to optimize treatment outcomes [88,89].

## 5. Laboratory Management of New BLBLI Agents

Microbiological laboratories play a critical role in monitoring the effectiveness of BLBLI combinations, as there is no complete cross-consensus in sensitivities and potencies between CZA-AVI, MER-VAB, and IMI-REL. Given this variability, minimum inhibitory concentrations (MICs) should be systematically determined for all three inhibitors to guide optimal therapeutic selection.

The introduction of immunochromatographic assays and molecular methodologies in many tertiary hospital laboratories has significantly improved the rapid detection of carbapenemase production prior to the availability of full antimicrobial susceptibility results [90]. Furthermore, the emergence of revolutionary rapid antimicrobial susceptibility testing (AST) technologies now allows for the determination of CAZ-AVI and MER-VAB MICs within 5–6 h, enabling faster implementation of targeted therapy [91]. This advancement necessitates a thorough understanding of the differences in in vitro activity of each BLBLI against various carbapenemases to enable more precise therapeutic decision-making. Identifying the specific carbapenemase type in a clinical isolate is essential, as CAZ-AVI, MER-VAB, and IMI-REL exhibit differential activity against class A, C, and D β-lactamases but lack activity against MBLs [3,4,10,21].

Continuous surveillance of susceptibility patterns by hospital microbiology laboratories is crucial for the early detection of emerging resistance to each inhibitor, as well as potential cross-resistance among them. This information is vital for implementing timely infection control measures and preventing further dissemination of resistant strains [92]. Regular screening of stool samples for the presence of KPC enzymes and general resistance to β-lactamase inhibitors is also an important aspect of antimicrobial stewardship, allowing for early identification of colonized patients who may require targeted infection prevention interventions [93].

In cases of treatment failure during BLBLI administration, it is imperative to obtain blood cultures and site-specific cultures to determine whether resistance has developed or if other factors, such as inadequate drug penetration at the infection site, have contributed to clinical failure. Studies have shown that microbiological failure associated with CZA-AVI is more frequently linked to the emergence of resistance in *K. pneumoniae* strains producing KPC-3. Fortunately, KPC-3-producing *K. pneumoniae* remains relatively uncommon in certain geographic regions, limiting its clinical impact [64]. Nonetheless, ongoing laboratory surveillance and resistance monitoring are essential to ensure the continued efficacy of these novel β-lactamase inhibitors.

## 6. Conclusions

Ceftazidime/avibactam, meropenem/vaborbactam, and imipenem/relebactam represent a new generation of β-lactam–β-lactamase inhibitor combinations that have redefined the treatment of KPC-producing Enterobacterales infections. All three demonstrate potent in vitro activity against KPC and other serine β-lactamase-producing MDR Enterobacterales, translating into high clinical cure rates in trials and practice. In real-world use, ceftazidime/avibactam and meropenem/vaborbactam have yielded excellent outcomes in KPC-*K. pneumoniae* infections, consistently outperforming historical regimens in survival. Imipenem/relebactam, while supported by more limited data so far, remains a promising alternative for KPC infections, especially when other options are unsuitable; however, it has shown promising efficacy against DTR *P. aeruginosa*. On the other hand, aztreonam/avibactam is promising for combating MBL-producing bacteria. It is essential to emphasize that these newer BLBLIs should be empirically administered only in cases where there is strong clinical suspicion or confirmed evidence of multidrug-resistant Gram-negative infections with limited treatment options. To mitigate the risk of resistance development, antimicrobial stewardship principles should be applied rigorously.

## Figures and Tables

**Table 1:** Comparison of the in vitro activity of the newer BLBLIs against carbapenem-resistant Gram-negative isolates [4,21,24,25].

Antimicrobial Agents	Enterobacterales			Carbapenem Resistant	
	KPC	MBL ^1^	OXA-48	*P. aeruginosa*	*A. baumannii*
Ceftazidime/avibactam	+	−	+	+ ^3^	−
Meropenem/vaborbactam	+	−	−	−	−
Imipenem/cilastatin/relebactam ^2^	+	−	−	+ ^3^	−
Aztreonam/avibactam	+	+	+	−	−

+ = active; − = inactive. ^1^ MBL = metallo-β-lactamases. ^2^ Imipenem/cilastatin/relebactam exhibits no activity against *Proteus* spp., *Providencia* spp., and *Morganella* spp. ^3^ Susceptibility rates may vary according to the resistance mechanism and geographical region.

**Table 2:** Dosage administration of novel β-lactam–β-lactamase inhibitor combination [27,28,29,30,52].

Drug	Dose	Renal Adjustment	CRRT
Ceftazidime/avibactam	2.5 g (2 g/0.5 g) q8h (infusion over 3 h)	CrCl > 50: 2.5 g q8h CrCl 31–50: 1.25 g q8h CrCl 10–30: 0.94 g q12h CrCl < 10: 0.94 g q48h CrCl 6–15 (±HD): 0.94 g q24h CrCl ≤ 5 (±HD): 0.94 g q48h	CVVH: 1.25 g q8h CVVHDF: 2.5 g q8h
Meropenem/vaborbactam	4 g (2 g/2 g) q8h (infusion over 3 h)	CrCl > 50: 4 g q8h CrCl 30–49: 2 g q8h CrCl 15–29: 2 g q12h CrCl < 15: 1 g q12h HD: 1 g q12h (AD)	No data
Imipenem/cilastatin/relebactam	1.25 g (0.5 g/0.5 g/0.25 g) q6h infusion 30 min	CrCl ≥ 90: 1.25 g q6h CrCl 60–89: 1 g q6h CrCl 30–59: 0.75 g q6h CrCl 15–29: 0.5 g q6h CrCl < Not recommended HD: 0.5 g q6h (AD)	No data
Aztreonam/avibactam *	2.67 g (2 g/0.67 g) loading dose, then 2 g (1.5 g/0.5 g) q6h (infusion 3 h)	CrCl > 50: 2.67 g × 1, then 2 g q6h CrCl > 30–50: 2.67 g × 1 then 1 g q6h CrCl > 15–30: 1.8 g × 1, then 0.9 g q8h CrCl ≤ 15: 1.33 g × 1, then 0.9 g q12h HD: 1.33 g × 1, then 0.9 g q12h (AD)	No data

AD, dose administered after dialysis; h, hours; CrCl, creatinine clearance; CRRT, continuous renal replacement therapy; CVVH, continuous venovenous hemofiltration; CVVHDF, continuous venovenous hemodiafiltration; HD, hemodialysis; q6h, every 6 h; q8h, every 8 h; q12h, every 12 h; q24h every 12 h; q48h, every 48 h. * Each vial contains 1.5 g aztreonam and 0.5 g avibactam.

**Table 3:** A comprehensive comparison of novel BLBLIs [3,4,5,6,7,8,27,28,29,30,31,32,33,34,35,36,37,38,39,40,41,42,43,44,45,46,47,48,49,50,58,59,60].

	Ceftazidime/ Avibactam	Aztreonam/ Avibactam	Meropenem/ Vaborbactam	Imipenem/ Relebactam
**Mechanism of action**	Diazabicyclooctane non-β-lactam–β-lactamase inhibitor	Diazabicyclooctane non-β-lactam–β-lactamase inhibitor	Cyclic boronic acid inhibitor	Diazabicyclooctane non-β-lactam–β-lactamase inhibitor, structurally related to avibactam, differing by the addition of a piperidine ring to the 2-position of the carbonyl group
**Spectrum**	Enterobacterales and *P. aeruginosa* producing ESBL, KPC, AmpC, and some class D enzymes (OXA-48) Not active against MBL, *Acinetobacter* spp., and no activity against anaerobes	Enterobacterales producing ESBL, KPC, AmpC, OXA-48, and MBL As active as aztreonam alone against *P. aeruginosa* and *A. baumannii*, including MBL-producing isolate	Enterobacterales producing ESBL, KPC, and AmpC Not active against OXA-48-like or MBL As active as meropenem alone against *P. aeruginosa*, *Acinetobacter* spp.	Enterobacterales and *P. aeruginosa* producing ESBL, KPC, AmpC, and porin mutations Diminished inhibitor activity against OXA-48 No activity against MBL, *A. baumannii*, *Proteus* spp., *Providencia* spp., and *Morganella* spp.
**Indications**	cUTI, cIAI, HAP/VAP, bacteremia associated with cUTI, cIAI, HAP/VAP, infections by Gram-negative pathogens with limited treatment options	cUTI, cIAI, HAP/VAP, infections by Gram-negative pathogens with limited treatment options	cUTI, cIAI, HAP/VAP, bacteremia associated with cUTI, cIAI, HAP/VAP, infections by Gram-negative pathogens with limited treatment options	HAP/VAP, bacteremia associated with HAP/VAP, infections by Gram-negative pathogens with limited treatment options
**Efficacy**	Enterobacterales: 75–80% *P.aeruginosa*: 50–85%	Enterobacterales: 75%	Enterobacterales: 60–75%	Enterobacterales and *P. aeruginosa*: 70%
**Comments**	Preferred agent in penicillin allergy Approved in pediatric patients (≥3 months)	Only agent active against MBL	High sodium load	Neurologic side effects, i.e., seizure

BLBLI, β-lactam–β-lactamase inhibitor combinations; cIAI, complicated intra-abdominal infections; cUTI, complicated urinary tract infections; ESBL, extended-spectrum β-lactamase; HAP, hospital-acquired pneumonia; KPC, Klebsiella pneumoniae carbapenemase; MBL, metallo-β-lactamases; OXA, oxacillinase; VAP, ventilator-associated pneumonia.

## Data Availability

No new data were created or analyzed in this study.

## Abbreviations

The following abbreviations are used in this manuscript:

AmpC	AmpC β-lactamase
AMR	Antimicrobial Resistance
ATM-AVI	Aztreonam/Avibactam
BSI	Bloodstream Infection
BLBLI	β-lactam–β-lactamase Inhibitor
CAZ-AVI	Ceftazidime/Avibactam
cIAI	Complicated Intra-abdominal Infection
CrCl	Creatinine Clearance
CRE	Carbapenem-Resistant Enterobacterales
CRRT	Continuous Renal Replacement Therapy
CPE	Carbapenemase-producing Enterobacterales
CVVH	Continuous Veno-Venous Hemofiltration
CVVHDF	Continuous Veno-Venous Hemodiafiltration
DBO	Diazabicycloocatane
DTR	Difficult-to-Treat Resistant
ELF	Epithelial Lining Fluid
ESBL	Extended-Spectrum β-Lactamase
EU/EAA	European Union/European Economic Area
GES	Guiana extended-spectrum β-lactamase
ICU	Intensive Care Unit
IMI-REL	Imipenem/Cilastatin/Relebactam
KPC	Klebsiella pneumoniae Carbapenemase
MBL	Metallo-β-lactamase
MDR	Multidrug-Resistant
MER-VAB	Meropenem/Vaborbactam
MIC	Minimum Inhibitory Concentration
NDM	New Delhi Metallo-β-lactamase
OXA	Oxacillinase (OXA-48-like)
PK/PD	Pharmacokinetics/Pharmacodynamics
RRT	Renal Replacement Therapy
VIM	Verona Integron-encoded Metallo-β-lactamase
